# Comprehensive analysis of the expression and prognosis for *CDCAs* in head and neck squamous cell carcinoma

**DOI:** 10.1371/journal.pone.0236678

**Published:** 2020-07-27

**Authors:** Zeng-Hong Wu, Ming Fang, Yan Zhou

**Affiliations:** 1 Department of Otorhinolaryngology, Union Hospital, Tongji Medical College, Huazhong University of Science and Technology, Wuhan, Hubei, China; 2 Department of Infectious Diseases, Union Hospital, Tongji Medical College, Huazhong University of Science and Technology, Wuhan, Hubei, China; Sapporo Ika Daigaku, JAPAN

## Abstract

Head and neck squamous cell carcinoma (HNSCC), a tumor included oral cavity, lips, larynx, oropharynx, and the nasopharynx et al. The cell division cycle-associated (*CDCA*) protein family (*CDCA1-8*) critical for normal cell function and cancer cell proliferation. We explored the mutation signatures and expression levels of various *CDCAs* in detail in HNSCC. A comprehensive bioinformatics analysis pipeline based on copy number and gene expressions data from patients with HNSCC in order to given new insights into the possible functions and distinct prognostics that underlie *CDCAs* regulation. We compared the transcriptional expression of *CDCAs* in HNSCC and found significantly elevated mRNA expression of *CDCA1-8* in HNSCC tissues across multiple datasets. We also found *CDCA5/6/8* are over-expressed both transcriptionally and translationally in patients with HNSCC. Our results suggested that that mRNA levels of *CDCA1/2/4/7* related to the prognosis and can be used as a new useful biomarker for predicting the survival of HNSCC patients. The top 5 *CDCAs* neighboring gene alterations in HNSCCs were found in *MYC*, *STAG1*, *RAD21*, *KLHL9* and *NDC80*. Multivariable Cox proportional hazard model also showed that CD8+ T cells were higher (*P*<0.05) in HNSCC-HPV-pos patients and that this was related to *CDCA1/2/3/4/5/7*. This study utilizes online tools to conduct specific gene analyses from free open databases, but our study requires more large-scale genomics research and basic research.

## Introduction

Head and neck squamous cell carcinoma (HNSCC), a tumor included oral cavity, lips, larynx, oropharynx, and the nasopharynx et al[1]. The tumor with a yearly incidence of over 650,000 new diagnosis and 90,000 decease worldwide[2]. The risk factors for HNSCC involve in smoking, alcohol drinking and virus infection, such as human papilloma virus (HPV)[3]. Unfortunately, there is insufficiency of symptoms at the early stage of the cancer, causing most patients with HNSCC to be diagnosed at the progressive stages. Consequently, the survival rate of 5-year is below 50% and patients that suffer from local recurrence and metastasis have an even lower survival rate of 35%[4]. When in the advanced stage, therapeutics can affect organ structures function that related to swallowing and speaking, leading to a decline in the patient's quality of life[5,6]. The occurrence of HNSCC is a complicated mechanism that involves multiple molecules. Ni *et al*[7] found that HPV and HPV16 DNA was detected in 26.4% and 71% of the 303 HNSCCs, respectively. Thus, prophylaxis against HPV infection may help reduce the incidence of this disease. A recent study proposed that zeste homolog 2 (*EZH2*) regulates epithelial-to-mesenchymal transition (EMT), metastasis and tumor invasion in HNSCC by regulating the *STAT3/VEGFR2* axis[8]. Valenti *et al*[9] reported that miR-205-5p's can impact genomic instability in HNSCC by selectively targeting the DNA damage response (DDR) genes *RAD17* and *BRCA1*. In spite of the advances that have been made in the past decades, which include combining chemotherapy, radiation, and surgery, many patients still experience tumors recurrences and metastasis even received treatment, which leads to therapeutic failure[10].

The cell division cycle-associated (*CDCA*) protein family (*CDCA1-8*) not only necessary for normal cell function, but also plays a key role in cancer cell proliferation. Some studies have highlighted that abnormal expression of cell cycle regulatory proteins may cause cancer. Phan *et al*[11] found that *CDCA3/5/8* are significantly higher in breast cancer tissue than control tissue, leading to a dramatic reduction in patient survival among breast cancer patients. A clinical trial that was now performed with castration resistant prostate cancer (CRPC) by a *CDCA1* peptide vaccination was found to effectively induce peptide-specific CTLs for CRPC patients[12]. In addition, siRNA-mediated knockdown of *CDCA1* in oral cavity carcinoma (OCC) tumor cells was found to induce a significant apoptotic response[13]. The *CDCA1* protein family is often co-expressed with many other cell cycle regulators, involving *CDC23/CDC7/CDC2/MCAK/MKI67* and topoisomerase II, to regulate tumor cell growth[14]. To date, the mechanism by which *CDCAs* are activated or deactivated in the development and progression of HNSCC still remains unclear. We explored the mutation signatures and expression levels of various *CDCAs* in detail using a comprehensive bioinformatics analysis pipeline based on copy number and gene expressions data from patients with HNSCC in order to offer more knowledge into the potential functions and distinct prognostics that underlie *CDCAs* regulation. We also discuss the opportunities and challenges in using these to derive clinical benefit for HNSCC patients.

## Methods and materials

### ONCOMINE database and Human Protein Atlas

The HNSCC mRNA expression data of *CDCAs* were obtained from the Oncomine[15], which is a database that involve 86,733 samples and 715 gene expression data sets. Oncomine as well the largest oncogene chip database as well as incorporated data mining database. This analysis was based on a number of prior HNSCC researches. The level of *CDCAs* was evaluated in HNSCC tissue and in control tissue. *P*<0.05 considered statistically significant. All the Data from Genomic Data Commons Data Portal. The Human Protein Atlas (HPA) is an online tool that included immunohistochemistry expression data for distribution and expression of proteins across 20 cancer tissues, 48 human normal tissues, 47 cell lines, and 12 blood cells[16]. We used immunohistochemistry images to directly compare protein expression of *CDCAs* among normal and cancer tissues.

### GEPIA dataset and UALCAN analysis

GEPIA[17] is an interactive online database which allowed users to found RNA seq expression data or samples based on the Genotype Tissue Expression projects (GTEx) and The Cancer Genome Atlas (TCGA). Meanwhile, GEPIA also offers customizable functions such as profiling based on pathological stage of cancer, type of cancer, survival analysis, correlation analysis and similar gene identification. UALCAN[18] is a website that helps analyze, integrate and discover cancer transcriptomic data and deep analyses of TCGA gene expression information. One of the portal’s highlight characteristic is that it can determined biomarkers or to perform *in silico* analysis of potential candidate genes of interest to assess expression in various subgroups, such as age, gender, race, and grade.

### Kaplan-Meier plotter and cBioPortal

Kaplan-Meier plotter[19] was used to predicted the prognostic significance of different *CDCAs* in HNSCC. The database includes RNA-seq information based on TCGA and GEO. By setting different parameters, different subgroups can explore including patients with various pathologies, treatment ways, and data sets. The cBioPortal[20] is a free asset that can download large-scale cancer genomics data sets encompassing 245 cancer researches. Using cBioPortal to explored *CDCAs* genetic alterations in *CDCAs*. An interaction network of the *CDCAs* and the co-expressed genes were also analyzed. GO and KEGG functions of *CDCAs* mutations and top 50 genes that were obviously linked to *CDCAs* mutations were performed via DAVID online tool.

### TIMER analysis

TIMER[21] is a useful tool for systematic found of immune infiltrates across different cancer types. Gene module can explore correlation among *CDCAs* and the abundance of immune infiltrates in HNSCC. The survival module was used to draw Kaplan-Meier plots for immune infiltrates and *CDCAs* for visualization of survival differences.

## Results

### High-expression of *CDCAs* family members

**Fig 1:**
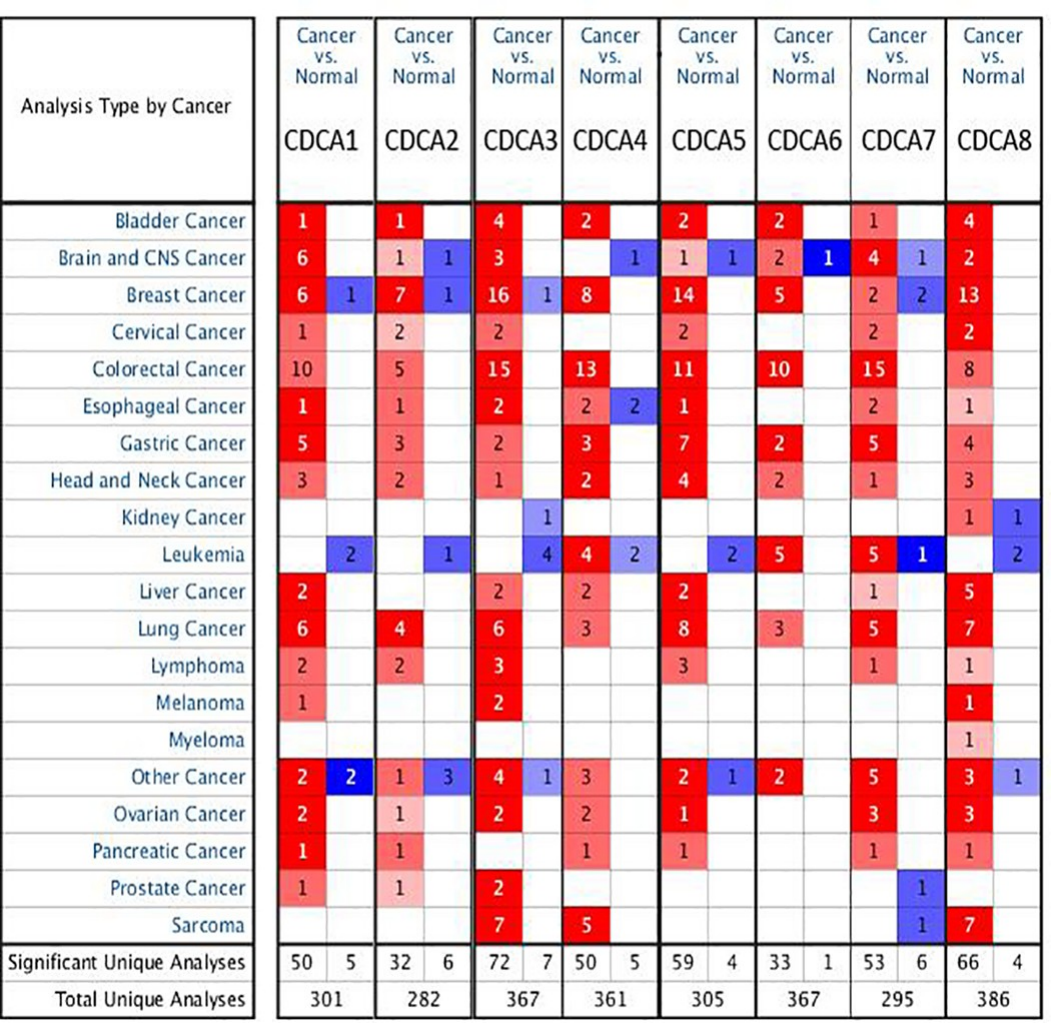
The transcriptional levels of CDCAs in cancers and normal samples and significantly higher mRNA expressions of CDCAs were found in HNSCC tissues in multiple datasets. (ONCOMINE database).

We first investigate the mRNA and protein expression of *CDCAs* using the ONCOMINE and HPA. We found obviously elevated expression of *CDCA1-8* in HNSCC tissues (Fig 1). According to the Peng statistics[22], *CDCA1* expression is 1.982-fold higher in OCC tissues compared to normal samples (*P* = 3.03E-9), Pyeon[23] observed 6.027-fold increase in *CDCA1* across multiple HNSCC cancer samples (*P* = 4.64E-7), and Sengupta[24] found 4.267-fold in HNSCC tissues (*P* = 1.22E-5, Table 1). Pyeon[23] observed 1.974-fold increase in *CDCA2* (*P* = 9.34E-6) and Sengupta[24] found a 2.490-fold increase in *CDCA2* (*P* = 1.70E-6). Pyeon[23] observed 1.926-fold increase in *CDCA3* (*P* = 4.16E-6). Data from Peng Head-Neck statistics[22] indicates that *CDCA4* is over-expressed in OCC tissues with a fold change of 1.580 (*P* = 3.76E-9), while Pyeon[23] observed 2.001-fold increase in *CDCA4* (*P* = 3.87E-10). In Peng statistics[22], *CDCA5* was found in the OCC tissues with a fold change of 1.764 (4.16E-12), Pyeon[23] observed 2.268-fold increase in *CDCA5* (*P* = 9.34E-6), Sengupta[24] found 2.055-fold increase in *CDCA5* (*P* = 7.02E-7) and Ye[25] observed a 2.553-fold increase of *CDCA5* in tongue tissue (*P* = 4.93E-9). Significant up-regulation of *CDCA6* was also found in HNSCC tissues. In Sengupta[24], *CDCA6* was found to high expressed with a fold change of 1.574 (*P* = 2.09E-5). According to Ye[25] statistics, *CDCA6* was high expressed with a fold change of 1.728 (*P* = 3.66E-6). Sengupta[24] showed a 2.402-fold increase in *CDCA7* (*P* = 1.22E-6). According to Giordano[26], *CDCA8* found a fold change of 1.515 (*P* = 4.63E-5). Similarly, Pyeon[23] statistics indicate that *CDCA8* with a fold change of 1.728 (*P* = 5.82E-7) and Peng statistics[22] observed a 1.607-fold in tumor samples (*P* = 1.41E-7).

**Table 1:** The significant changes of CDCA expression in transcription level between different types of HNSCC and normal tissues (Oncomine database).

CDCAs	Type of HNSCC† Cancer versus Normal HNSCC Tissue	Fold Change	*P* Value	t Test	Source and/or Reference
CDCA1	Oral Cavity Squamous Cell Carcinoma	1.982	3.03E-9	6.934	Peng Head-Neck statistics[22]
	Multi-cancer	6.027	4.64E-7	7.030	Pyeon Multi-cancer[23]
	Nasopharyngeal Carcinoma	4.267	1.22E-5	5.894	Sengupta Head-Neck Statistics[24]
CDCA2	Multi-cancer	1.974	9.34E-6	5.314	Pyeon Multi-cancer[23]
	Nasopharyngeal Carcinoma	2.490	1.70E-6	6.549	Sengupta Head-Neck Statistics[24]
CDCA3	Multi-cancer	1.926	4.16E-6	5.716	Pyeon Multi-cancer[23]
CDCA4	Oral Cavity Squamous Cell Carcinoma	1.580	3.76E-9	6.636	Peng Head-Neck statistics[22]
	Multi-cancer	2.001	3.87E-10	10.331	Pyeon Multi-cancer[23]
CDCA5	Oral Cavity Squamous Cell Carcinoma	1.764	4.16E-12	8.049	Peng Head-Neck statistics[22]
	Multi-cancer	2.268	9.34E-6	7.206	Pyeon Multi-cancer[23]
	Nasopharyngeal Carcinoma	2.055	7.02E-7	6.641	Sengupta Head-Neck Statistics[24]
	Tongue Squamous Cell Carcinoma	2.553	4.93E-9	7.693	Ye Head-Neck statistics[25]
CDCA6	Nasopharyngeal Carcinoma	1.574	2.09E-5	4.697	Sengupta Head-Neck Statistics[24]
	Tongue Squamous Cell Carcinoma	1.728	3.66E-6	6.167	Ye Head-Neck statistics[25]
CDCA7	Nasopharyngeal Carcinoma	2.402	1.22E-6	5.530	Sengupta Head-Neck Statistics[24]
CDCA8	Thyroid Gland	1.515	4.63E-5	7.129	Giordano Thyroid Statistics[26]
	Multi-cancer	1.822	5.82E-7	9.003	Pyeon Multi-cancer[23]
	Oral Cavity Squamous Cell Carcinoma	1.607	1.41E-7	5.759	Peng Head-Neck statistics[22]

†HNSCC: head and neck squamous cell carcinoma.

**Fig 2:**
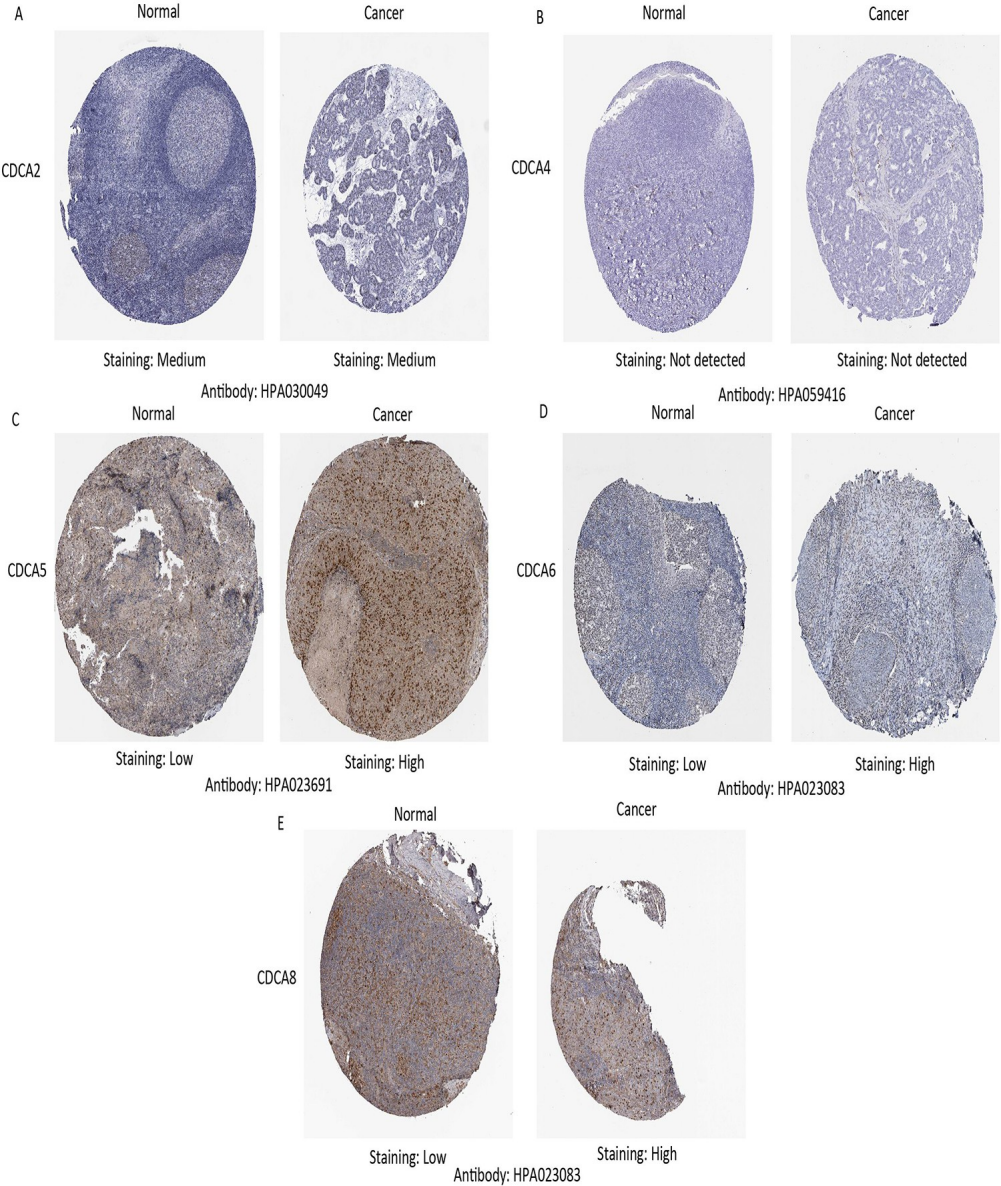
Representative immunohistochemistry images of distinct CDCAs family members in HNSCC tissues and normal tissues (Human Protein Atlas database).

We next analyzed the protein expression of *CDCAs* and the result indicated low protein expression of *CDCA5/6/8* in normal tissues and high protein expression in tumor tissues. In addition, results also indicate medium expression of *CDCA2* in normal tissues and high expression in tumor tissues. Meanwhile, we observed no protein expression of *CDCA4* in either normal or HNSCC tissues (HPA database missed CDCA1/3/7 data, Fig 2). Overall, our results suggest that *CDCA5/6/8* are over-expressed both transcriptionally and translationally in patients with HNSCC.

### Clinical subgroup analysis

**Fig 3:**
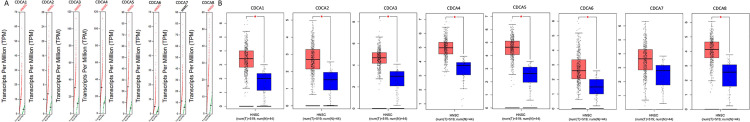
The box plot expression of CDCAs in HNSCC (GEPIA database).

**Fig 4:**
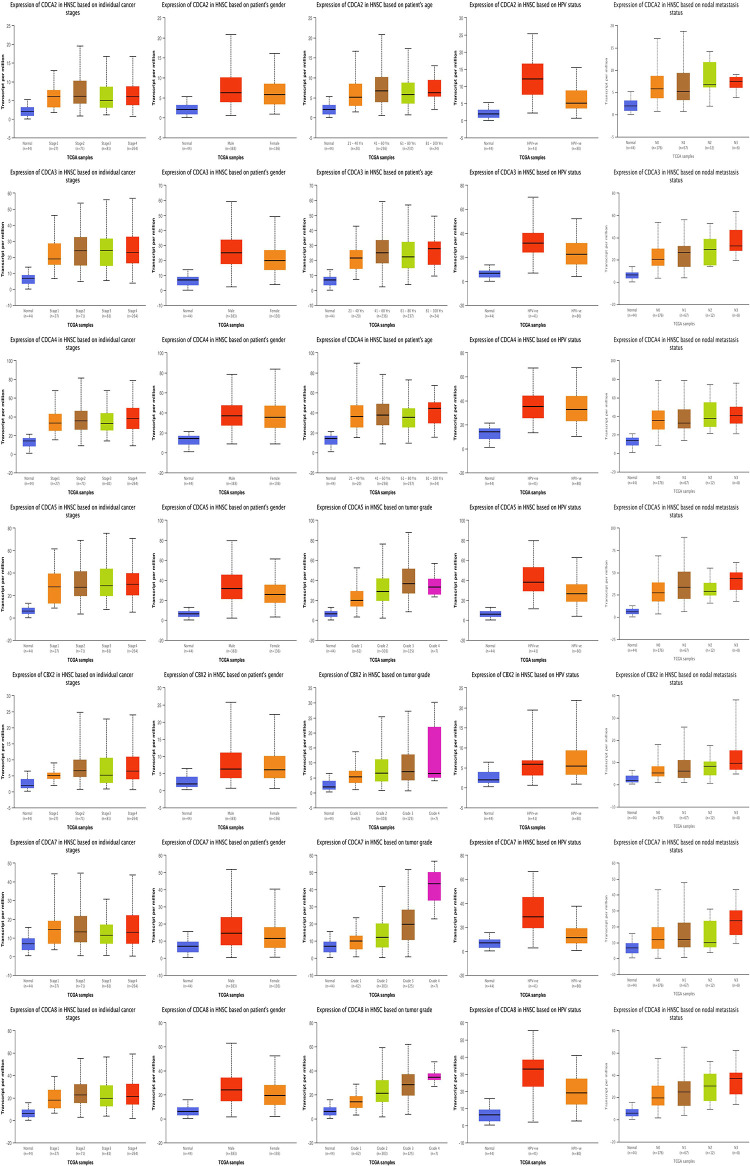
Boxplot showing relative expression of CDCAs in subgroups of patients with HNSCC, stratified based on gender, age, HPV status, gender and tumor grade (UALCAN).

We first using the GEPIA dataset to compared the expression of *CDCAs* among cancer and normal tissues. Our results indicate that the *CDCA1/2/3/4/5/6/8* are significantly higher in HNSCC tissues (Fig 3). Next, we performed subgroup analysis of multiple clinical pathological features using the TCGA database. Subgroup analysis by age, indicated that transcriptional levels of *CDCAs* were higher in HNSCC patients when compared to healthy individuals. Additionally, subgroup analysis by HPV status analysis; gender subgroup, and tumor grade demonstrated that *CDCAs* were significantly higher in HNSCC patients across all subgroups (Fig 4).

### Prognostic analysis

**Fig 5:**
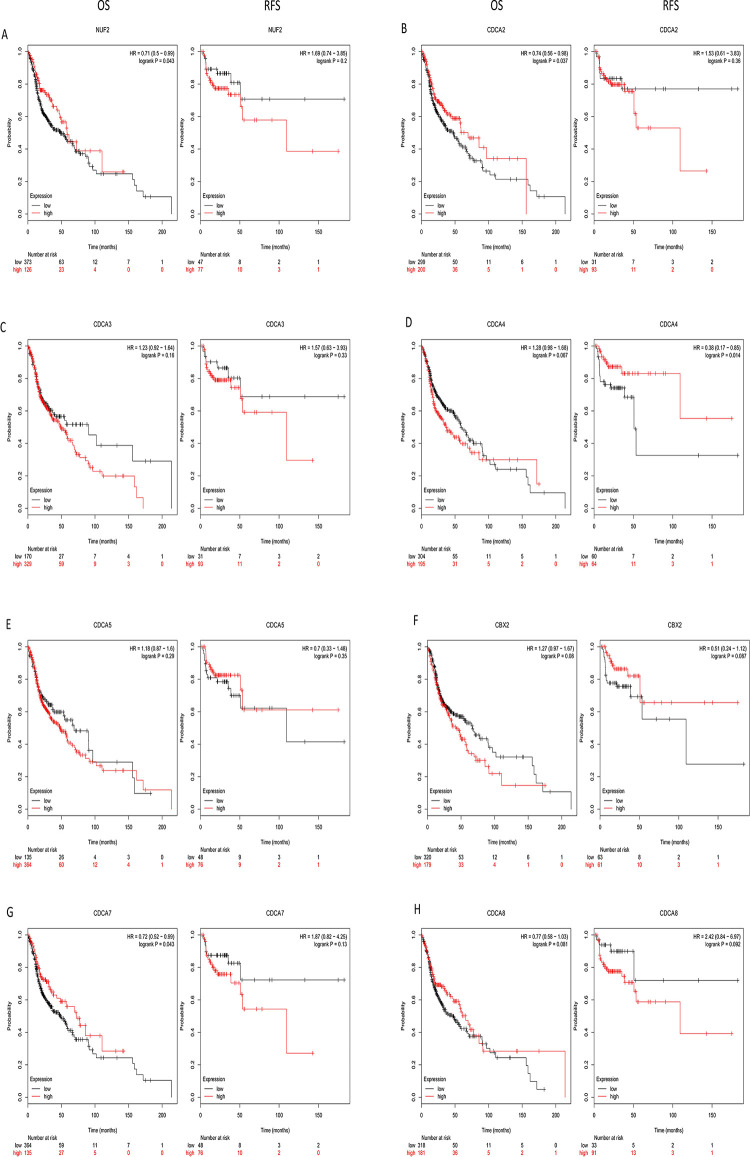
The prognostic value of mRNA level of CDCAs in HNSCC patients (Kaplan-Meier Plotter database).

Next, we tried to explore the prognostic significance of *CDCAs* in HNSCC patients, data for which was obtained from publicly available online datasets. The results are shown in Fig 5, which indicate that higher expression of *CDCA4* (HR = 0.38, 95% CI: 0.19–0.85, *P* = 0.014) was related to longer relapse free survival (RFS). Higher expression of *CDCA1* (HR = 0.71, 95% CI: 0.50–0.99, *P* = 0.043), *CDCA2* (HR = 0.74, 95% CI: 0.56–0.99, *P* = 0.037) and *CDCA7* (HR = 0.72, 95% CI: 0.52–0.99, *P* = 0.043) was also related to longer overall survival (OS). These results suggest that the levels of *CDCA1/2/4/7* may play a key role in HNSCC prognosis.

### Function analysis of *CDCAs* in HNSCC

**Fig 6:**
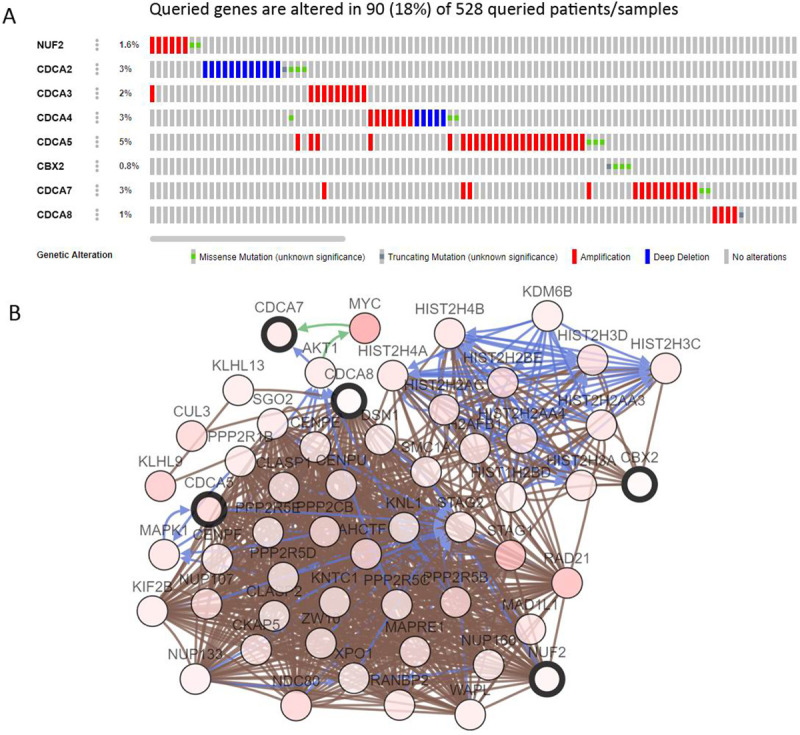
CDCAs gene expression and mutation analysis in HNSCC (cBioPortal database). A. CDCAs gene expression and mutation analysis; B. The network for CDCAs and the 50 most frequently altered neighbor genes.

We explored *CDCAs* alterations and networks using the cBioPortal. 50 neighboring genes that were found to be significantly linked to *CDCAs* mutations. Among the 528 HNSCC tumor samples that were sequenced, genetic alterations were found in 90 samples with a mutation rate of 18%. *CDCA5* was ranked as the most mutated gene among *CDCAs* with mutation rates of 5%. We also showed the network for *CDCAs* and the 50 most frequently altered neighboring genes (Fig 6). The top 5 *CDCAs* neighboring gene alterations in HNSCCs were found in *MYC*, *STAG1*, *RAD21*, *KLHL9* and *NDC80* (Table 2).

**Table 2:** The top 5 type and frequency of *CDCAs* neighbor gene alterations in HNSCC (cBioPortal).

Gene Symbol	Amplification	Mutation	Total Alteration
MYC	12.1	1.2	13.2
STAG1	8.9	1.0	9.7
RAD21	8.3	1.0	9.3
KLHL9	0.8	0.6	7.3
NDC80	5.0	0.8	6.0

Next, we analyzed the functions of *CDCAs* and these 50 genes using GO and KEGG (S1 Appendix). GO analysis indicate that changes in biological processes included enrichment in sister chromatid cohesion, cell division, mitotic nuclear division, gene silencing by RNA, and protein sumoylation among others. Molecular function was mainly enriched in protein heterodimerization activity, microtubule plus-end binding, protein phosphatase type 2A regulator activity, nucleocytoplasmic transporter activity, and protein binding. Changes in cell component were largely enriched in condensed chromosome kinetochore, chromosome, centromeric region, kinetochore, cytosol, nucleosome and others. Pathway enrichment analysis according to KEGG was mainly enriched in PI3K-Akt and AMPK signaling pathway, endometrial cancer, acute myeloid leukemia, colorectal cancer, central carbon metabolism in cancer, transcriptional misregulation in cancer, and chronic myeloid leukemia.

### Immune infiltrates of *CDCAs* in HNSCC

**Fig 7:**
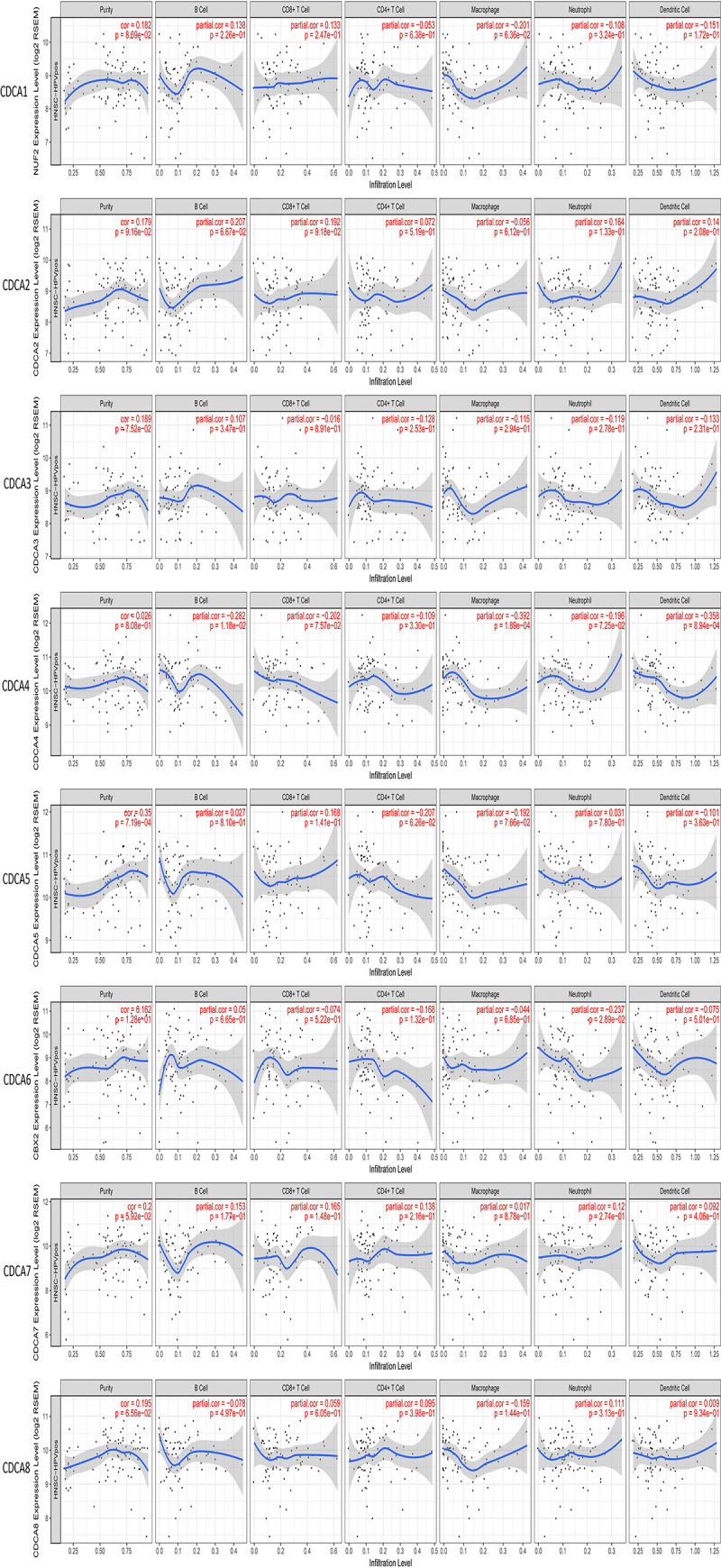
Correlation between CDCAs in HNSCC expression and abundance of immune infiltrates was statistically significant (*P*<0.05). (TIMER database).

**Fig 8:**
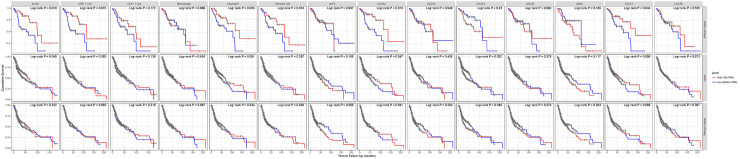
The difference cumulative survive between HNSCC, HNSCC-HPV-pos and HNSCC-HPV-neg and in HNSCC-HPV-pos group showed that B cells, CD8+ T cells and neutrophil cells of immune infiltrates statistically significant (*P*<0.05) of CDCAs.

There is a statistically significant correlation between *CDCAs* expression in HNSCC and abundance of immune infiltrates (*P*<0.05, Fig 7). We explored the difference in cumulative survival between HNSCC, HNSCC-HPV-pos and HNSCC-HPV-neg and found that the HNSCC-HPV-pos subgroup showed significantly higher B cells, CD8+ T cells and neutrophil immune infiltrates, (*P*<0.05) which was related to *CDCAs* levels. This indicates that these immune cell infiltrations significantly affect prognosis. Therefore, it is worth further researching and exploring this association (Fig 8). Multivariable Cox proportional hazard model also showed that CD8+ T cells immune infiltrates were significant higher (*P*<0.05) in HNSCC-HPV-pos patients and that this was related to *CDCA1/2/3/4/5/7* Table 3.

**Table 3:** Multivariate survival model analysis based on TIMER online tool (HNSCC-HPVpos).

Clinicopathologic variable	coef	HR	95% CI L	95% CI U	p-Value	sig
Age	-0.012	9.880E-01	0.908	1.076E+00	0.789	
Gender Male	-0.153	8.580E-01	0.127	5.803E+00	0.875	
Race Black	19.439	2.767E+08	0	Inf	0.999	
Race White	18.555	1.145E+08	0	Inf	0.999	
Stage II	17.660	4.671E+07	0	Inf	0.998	
Stage III	14.808	2.700E+06	0	Inf	0.999	
Stage IV	15.707	6.626E+06	0	Inf	0.999	
Purity	-0.462	6.300E-01	0.010	3.942E+01	0.827	
B cells	12.517	2.730E+05	0	1.380e+16	0.320	
CD+ 8 T cell	-24.639	0	0	0	0.002	**
CD4+ T cells	-10.185	0	0	2.371e+01	0.135	·
Macrophages	17.151	2.808E+07	0.005	1.589E+17	0.134	
Neutrphils	-10.292	0	0	2.450E+01	0.135	
Dendritic	8.031	3.074E+03	0.014	6.843E+08	0.201	
NUF2	2.975	1.960E+01	2.702	1.421E+02	0.003	**
CDCA2	-1.315	2.680E-01	0.080	9.010E-01	0.033	*
CDCA3	-2.501	8.200E-02	0.018	3.680E-01	0.001	**
CDCA4	1.834	6.260E+00	1.469	2.668E+01	0.013	*
CDCA5	2.282	9.800E+00	1.748	5.496E+01	0.009	**
CBX2	-0.300	7.410E-01	0.343	1.598E+00	0.444	
CDCA7	-1.141	3.190E-01	0.135	7.550E-01	0.009	**
CDCA8	0.541	1.718E+00	0.151	1.950E+01	0.663	

P-value Significant Codes: 0 ≤ ***< 0.001 ≤ **< 0.01 ≤ *< 0.05 ≤·< 0.1.

## Discussion

Though certain *CDCAs* have been shown to play a critical role in tumor, the specific roles of *CDCAs* in HNSCC remains unclear. Thus, we first explored the mutational, gene expression, and prognostic landscape of various *CDCAs* in patients with HNSCC. We found higher mRNA expression across all *CDCAs*, and the expression of *CDCAs* was significantly linked to patients’ individual cancer stages. Moreover, we explored the immune status of HNSCC patients which can potentially help guide the development of novel therapies and to improve response to immunotherapy.

A growing number of studies have shown that *CDCAs* are highly expressed in tumors and have a role in regulating tumor cell cycle, promoting tumor cell proliferation, and reducing tumor cell apoptosis, which results in poor prognosis. *CDCA1*, also known as *NUF2*, codes for a protein that is essential for nuclear division and microtubule stabilization[27]. Tokuzum *et al*[27] reported that *CDCA1*-specific siRNA inhibits the cell proliferation of *WM115* and *SKMEL2* cells, but does not reduce the invasion activity or migration in malignant melanoma patients. Tomita *et al*[28] demonstrated that the existence of *CDCA1*-specific Th cell responses in HNSCC patients underline the potential utility of *CDCA1*-LPs for propagation of both *CDCA1*-specific CTLs and Th cells. Similarly, Kaneko *et al*[29] found that knockdown of *CDCA1* and *KNTC2* genes in colorectal cancer cells better inhibits tumor cell growth. Our results show that *CDCA1* is highly expressed in HNSCC tissues, and *CDCA1* is significantly correlated to patients’ survival and abundance of immune infiltrates. Moreover, our cumulative survival analyses show that CD8+ T cell immune infiltrates significantly affect the prognosis of these patients. Thus, it is worth further exploring this association.

*CDCA2* is a nuclear protein that binds to protein phosphatase 1γ (PP1γ) and participated in DNA damage during cell cycle[30]. Moreover, *CDCA2* modulates phosphorylation of the primary mitotic histone H3 in a PP1-dependent manner[31]. Some studies indicated that *CDCA2* act for a very powerful prognostic marker for poor patient survival and malignancy in cancers such as neuroblastoma, lung adenocarcinoma, and oral squamous cell carcinoma tissue[32–34]. A recent study found that overexpression of *CDCA2* may target *CCND1* to promote colorectal cancer cell proliferation and tumorigenesis via activation of the *PI3K/AKT* pathway[35]. Interestingly, in our analysis of 50 neighbor genes that were significantly related to *CDCAs* mutations, the KEGG results showed a high enrichment of genes involved in the *PI3K-Akt* and *AMPK* signaling pathway. Thus, our study provides critical information that can be utilized for future studies.

*CDCA3* is part of the SKP1-Cullin-RING-F-box (SCF) ubiquitin ligase (E3) complex, which degrades the endogenous cell cycle inhibitor WEE1, thereby regulating cell cycle[36]. *CDCA3*, through regulation by specificity protein 1 (*SP1*) and hypomethylation of its gene body, affects gastric cancer (GC) cell proliferation and invasion[37]. In addition, *CDCA3* activated the Ras signaling pathway to facilitate cell proliferation *in vitro* and *in vivo* in GC cells[38]. Another study also found that HoxB3 can bind to the *CDCA3* promoter region and transactivate *CDCA3* expression to induce prostate cancer progression[39]. Our results show that HNSCC tissue highly express *CDCA3*. To date, however, no studies have investigated the connection between HNSCC and *CDCA3* and more research is needed.

*CDCA4*, also known as HEPP/SEI-3/TRIP-Br3 is a target gene of transcription factor E2F, was discovered in 2001 and has shown to be related to the regulation of genes regulating the growth and differentiation of hematopoietic stem and progenitor cells[40]. Xu *et al*[41] found that *CDCA4* enhanced proliferation and reduced apoptosis in the MCF‑7/ADM breast cancer cells *in vitro*. A recent study also suggested that *CDCA4* may be involved in regulating triple negative breast cancer (TNBC) progression[42]. Results from our study indicate that *CDCA1/2/4/7* may serve as novel biomarkers for prediction of HNSCC patients’ survival. *CDCA5* is a critical regulator of sister chromatid condensation and separation during cell division[43]. *CDCA5* could promote proliferation, migration, invasion, apoptosis resistance and decrease chemosensitivity to cisplatin in esophageal squamous cell carcinoma (ESCC) cells[44]. Moreover, *CDCA5* was shown to be upregulated in hepatocellular carcinoma (HCC) tissues compared to paracancerous tissues, is negatively correlated with patient survival and associated with cell abnormalities via upregulation of the AKT pathway[45]. *CDCA6* also known as *CBX2*, encodes a component of the polycomb multiprotein complex. *CDCA6* depletion abrogated cell viability and induced caspase 3-mediated apoptosis in metastatic prostate cancer cell lines[46]. One study also found that *CDCA6* upregulation and amplification was significantly related to lower overall survival and metastatic progression across many cancer types[47]. While our study shows high expression of *CDCA6* in HNSCC tissues, though there is a paucity of studies in literature that have investigated this connection. Thus, there is a need to conduct research on the role of *CDCA6* in HNSCC. *CDCA7*, also known as *JPO1*, is considered to be a c-Myc target gene that is involved in c-Myc-mediated cell transformation[48]. One study found that depletion of *CDCA7* extremely minimize the tumorigenicity and colonization capacities of TNBC cells *in vivo*[49]. Jenness *et al* reported that the *HELLS-CDCA7* complex possesses nucleosome remodeling activity[50]. Another study discovered a role for *CDCA7* in Centromeric Instability and Facial Anomalies syndrome, a life-threatening immunodeficiency[51]. In addition, AKT signaling to *CDCA7* could alter MYC-dependent growth and transformation, contributing to tumorigenesis[52].

*CDCA8*, also known as Borealin/DasraB, encodes a component of the chromosomal passenger complex and is essential for chromatin-induced microtubule stabilization and spindle formation[53]. One study also reported that *CDCA8* was significantly linked to poor prognosis in patients with cutaneous melanoma[54], breast cancer[55], colorectal cancers[56] and lung cancer[57]. Our results suggest that *CDCA5/6/8* are higher expressed in patients with HNSCC, both transcriptionally and translationally. Overall, the function and pathways of *CDCAs* and their 50 frequently altered neighboring genes showed that these genes were mainly enriched in changes in cell division, mitotic nuclear division, protein binding and other cell functions. KEGG pathway analysis showed an enrichment in PI3K-Akt and AMPK signaling pathway, as well as some cancers and cancer-related signaling pathway. Thus, modifications to *CDCAs* is associated with post-transcriptional regulation, which is largely linked to protein translation.

To date, no studies have investigated the role of *CDCAs* and the connection between tumor infiltrating immune cells and HNSCC. We first explored the difference between cumulative survival between HNSCC, HNSCC-HPV-pos and HNSCC-HPV-neg tumors and found that HNSCC-HPV-pos group had a significantly higher infiltration of B cells, CD8+ T cells and neutrophil cells (*P*<0.05), which was positively related to CDCAs expression. This indicates that immune cells may have a significant effect on the prognosis of this disease. Therefore, it is worth further investigation in subsequent studies. There were several limitations, one being that all the data in our study was based on online free databases. Additionally, our study does not provide precise clinical information. Hence, more studies are needed to prove our findings. Another limitation is that we did not assess the possible therapeutic and diagnostic roles of *CDCAs* as the histological types of HNSCC as well as the multiple anatomical sites of the cancer varies widely. Thus, future studies are needed. Finally, we were incapable to contrast the differences in function of *CDCAs* among HPV-positive and HPV-negative in HNSCC due to insufficient data, though we plan to investigate this in the future.

## Conclusion

Our results indicate that *CDCAs* play a key role in the HPV-pos HNSCC patients. This study made use of online free tools to perform target gene analyses on HNSCC from open databases, which enables more genomics research and subsequent functional exploration.

## Supporting information

S1 Appendix Functions and pathways of CDCAs and their 50 frequently altered neighbor genes were analyzed by GO and KEGG in DAVID online database. (DAVID database).
(XLSX)
